# Clinical Significance of TAPSE/PASP Ratio in Risk Stratification for Aortic Stenosis Patients Undergoing Transcatheter Aortic Valve Replacement

**DOI:** 10.3390/jcdd12120468

**Published:** 2025-11-29

**Authors:** Simina Mariana Moroz, Alina Gabriela Negru, Silvia Luca, Daniel Nișulescu, Mirela Baba, Darius Buriman, Ana Lascu, Daniel Florin Lighezan, Ioana Mozos

**Affiliations:** 1 Center for Advanced Research in Cardiovascular Pathology and Hemostaseology, Victor Babeş University of Medicine and Pharmacy, 300041 Timișoara, Romania; simina.moroz@umft.ro (S.M.M.);; 2 Doctoral School Medicine-Pharmacy, Victor Babeş University of Medicine and Pharmacy, 300041 Timișoara, Romania; drmirela88@yahoo.com (M.B.);; 3 Cardiology Department, Victor Babeş University of Medicine and Pharmacy, 300041 Timișoara, Romania; 4 Institute of Cardiovascular Diseases Timișoara, 300310 Timișoara, Romania; 5 Research Center of the Institute of Cardiovascular Diseases Timișoara, 300310 Timișoara, Romania; 6 Department of Histology, Faculty of Medicine, Vasile Goldiș Western University of Arad, 310025 Arad, Romania; 7 Center for Translational Research and Systems Medicine, Victor Babeş University of Medicine and Pharmacy, 300041 Timișoara, Romania; 8 Department of Functional Sciences-Pathophysiology, Victor Babeş University of Medicine and Pharmacy, 300041 Timișoara, Romania; 9 Department of Internal Medicine I-Medical Semiotics I, Victor Babeş University of Medicine and Pharmacy, 300041 Timișoara, Romania

**Keywords:** TAPSE/PASP ratio, transcatheter aortic valve replacement, aortic stenosis, right ventricular function, atrial fibrillation, prognostic marker, risk stratification

## Abstract

Aortic stenosis (AS), a progressive valvular disease that results in increasing left ventricular (LV) afterload, leads to ventricular dysfunction and heart failure if left untreated. Transcatheter aortic valve replacement (TAVR) has emerged as a minimally invasive and effective alternative to surgical replacement, especially in elderly or high-risk patients. **Objectives**: The present study aims to assess the influence of the tricuspid annular plane systolic excursion (TAPSE)/pulmonary systolic arterial pressure (PASP) ratio on clinical outcomes in patients with aortic stenosis undergoing TAVR and offer valuable insights into patient selection and tailored management strategies for individuals undergoing TAVR. **Methods**: A retrospective analysis was conducted on 100 patients with AS who underwent TAVR, included in two distinct groups based on their median TAPSE/PASP ratio. **Results**: Patients were divided according to their median TAPSE/PASP ratio into two groups. Those with lower TAPSE/PASP ratios had a higher incidence of post-procedural atrial fibrillation (AF) (48% vs. 28%, *p* = 0.0404), lower left-ventricular ejection fraction (LVEF) (41.06% vs. 49.50%, *p* < 0.0001), a more pronounced inflammatory and hematologic response, and longer hospitalization. Receiver-operating characteristic (ROC) analysis demonstrated modest but significant discrimination rather than high sensitivity or specificity for postprocedural arrhythmias, particularly atrial fibrillation. **Conclusions**: TAPSE/PASP should be regarded as a clinically useful risk-stratification marker in patients with AS undergoing TAVR, enabling the identification of high-risk patients and optimizing peri-procedural management.

## 1. Introduction

Aortic stenosis (AS) is a progressive valvular disease characterized by aortic valve narrowing, which leads to increased left ventricular (LV) afterload, reduced cardiac output, and heart failure if left untreated [1]. Transcatheter aortic valve replacement (TAVR) has emerged as a minimally invasive alternative to surgical aortic valve replacement, offering a viable solution particularly for high-risk and elderly patients [2,3].

Despite advancements in TAVR procedures, pre-existing conditions such as atrial fibrillation (AF) pose significant challenges and negatively impact postprocedural outcomes. Pre-existing atrial fibrillation (AF)—paroxysmal, persistent, or permanent—remains a frequent comorbidity and an adverse prognostic factor. Atrial fibrillation, the most common sustained cardiac arrhythmia, with a particularly high prevalence in elderly patients with AS, is associated with increased procedural risks, heightened susceptibility to postprocedural complications, and prolonged recovery, emphasizing the need for comprehensive risk assessment and tailored management strategies to optimize results in affected individuals [3,4,5]. The coexistence of AF and AS presents significant clinical challenges, contributing to increased morbidity and mortality caused by the loss of atrial contraction, reduced LV filling, reduced cardiac output, and exacerbating cardiac instability. Moreover, AF predisposes to a higher risk of stroke and systemic thromboembolic events, particularly in patients with compromised ventricular function [6]. Among patients undergoing TAVR, AF is associated with prolonged hospitalization, higher rates of heart failure, and an increased risk of stroke [7]. The right ventricular (RV) function is an important determinant of outcomes in AS patients undergoing TAVR. Given the intricate relationship between RV contractility, pulmonary pressures, and hemodynamic stability, accurate assessment of RV performance is essential for risk stratification and optimal periprocedural management [8].

Among the key echocardiographic parameters used to evaluate RV function, tricuspid annular plane systolic excursion (TAPSE) serves as a direct measure of RV longitudinal systolic function, while pulmonary systolic arterial pressure (PASP) provides insights into pulmonary hemodynamics and RV afterload. The TAPSE/PASP ratio has gained recognition as a valuable prognostic marker, helping to identify patients at higher risk for postprocedural complications, including heart failure progression and prolonged hospitalization. By integrating RV function assessment into preprocedural evaluation, clinicians can tailor management strategies, refine patient selection criteria, and optimize interventional outcomes in AS patients undergoing TAVR. Right ventricular (RV) dysfunction is a common and often underestimated condition in patients with AS. The increased afterload due to chronic pressure overload from AS and pulmonary hypertension leads to RV remodeling and dysfunction [9,10,11,12]. Pulmonary arterial hypertension, frequently observed in this population, exacerbates RV failure and negatively impacts exercise capacity and survival [13]. The interaction between AF and RV dysfunction further complicates the hemodynamic status, leading to elevated right atrial pressures, impaired ventricular interdependence, and ultimately, heart failure [14].

RV function can be effectively assessed using echocardiographic parameters, with TAPSE serving as a reliable marker of longitudinal RV contraction [15]. However, TAPSE alone may not provide a comprehensive evaluation, requiring the use of the TAPSE/PASP ratio, which incorporates pulmonary pressures to better reflect RV adaptation to load [16,17]. Clinically, values > 0.32 mm/mmHg indicate preserved coupling efficiency, whereas values < 0.32 mm/mmHg identify impaired coupling and a higher risk [16]. Elevated PASP and reduced TAPSE contribute to right-atrial dilatation and increased atrial pressure, providing a substrate for AF development. Incorporating the TAPSE/PASP ratio into preprocedural evaluation could therefore refine patient selection and optimize hemodynamic management before and after TAVR [17,18,19].

A low TAPSE/PASP ratio indicates an inability of the RV to compensate for increased afterload and is associated with worse clinical outcomes in TAVR patients [18,19]. The evaluation of RV function should be considered alongside the assessment of pulmonary artery (PA) performance, as the RV and PA operate as a unitary and integrated cardiopulmonary system. This system is characterized by two primary components: the contractile capacity of the RV and the resistance imposed by the PA, commonly referred to as RV afterload. Under physiological conditions, these elements maintain a state of synchrony, termed RV-PA coupling, which facilitates the efficient transfer of energy from the RV to the pulmonary vessels [20,21]. TAPSE is a key echocardiographic parameter that quantifies the longitudinal movement of the lateral tricuspid annulus during RV systole. The measurement expressed in millimeters or centimeters is obtained using M-mode echocardiography. A TAPSE measurement of less than 17 mm (1.7 cm) is considered abnormal, suggesting RV dysfunction. In conclusion, the TAPSE/PASP ratio serves as an indicator used to evaluate RV contractility and its adaptation to pressure overload [20]. Moreover, the TAPSE/PASP ratio has been suggested as a marker for ventricular-atrial coupling in patients with pulmonary arterial hypertension (PAH) and to predict right ventricular failure and mortality in patients with PAH [5,21]. The 2022 ESC/ERS guidelines for the diagnosis and treatment of pulmonary hypertension acknowledge the TAPSE/PASP ratio as a supplementary echocardiographic indicator relevant to PAH assessment [22]. The comprehensive risk assessment of 1-year mortality in PAH can be evaluated by the TAPSE/PASP ratio as follows: <0.32 mm/mmHg low risk (<5%), 0.19–0.32 mm/mmHg intermediate risk (5–20%) and <0.19 mm/mmHg high risk (>20%) [22,23]. The TAPSE/PASP ratio is gaining recognition as a crucial prognostic marker for TAVR patients with AF. Studies have demonstrated that a lower TAPSE/PASP ratio is independently associated with increased all-cause mortality, hospital readmissions, and a higher likelihood of requiring post-TAVR inotropic support. Identifying patients with a compromised preprocedural TAPSE/PASP ratio can assist in risk assessment and guide preprocedural management approaches [24,25].

Moreover, patients with impaired RV function often exhibit signs of right-sided heart failure, including peripheral edema, hepatic congestion, and reduced exercise tolerance. These clinical signs, coupled with echocardiographic findings, require a multidisciplinary approach to optimize pre-TAVR hemodynamics through diuretic therapy, afterload reduction, and careful volume management [17,26].

The primary objective of this study is to assess the influence of lower TAPSE/PASP ratios on both preprocedural and postprocedural outcomes in patients with AS undergoing TAVR. By stratifying patients into two groups based on their median TAPSE/PASP values, the study aims to identify potential risk factors contributing to poor clinical outcomes and explore targeted strategies to enhance RV function before the procedure. This study aims to determine how a reduced TAPSE/PASP ratio influences peri-procedural outcomes in patients with severe AS undergoing TAVR and to assess its prognostic role for arrhythmic events. This approach aims to provide valuable insights into optimizing preprocedural management, improving patient prognosis, and refining therapeutic interventions for those with RV dysfunction.

## 2. Materials and Methods

### 2.1. Study Population

A retrospective analysis was conducted on a cohort of 100 patients (52 men and 48 women) with severe AS, who underwent TAVR between 2020 and 2023 in the Institute of Cardiovascular Diseases Timișoara, Romania. The study was approved by the Ethics Committee for Scientific Research of the “Victor Babes” University of Medicine and Pharmacy (Nr. 59/18.12.2019 rev 202424).

### 2.2. Inclusion and Exclusion Criteria

The inclusion criteria for this study required participants to be 65 years or older, with a focus on the elderly, a population at higher risk for aortic stenosis-related complications. Eligible patients had a confirmed diagnosis of severe AS, characterized by an aortic valve area (AVA) of ≤1.0 cm^2^, necessitating TAVR intervention. Only individuals with comprehensive preprocedural and postprocedural echocardiographic and laboratory data were included.

The exclusion criteria were designed to eliminate patients with conditions that could significantly impact outcomes or confound the analysis. Patients were excluded due to significant concomitant mitral or tricuspid valve disease, severe right ventricular failure unrelated to AS, active infection, or incomplete echocardiographic data. Individuals with a history of prior cardiac surgery were excluded to ensure a uniform study population, thereby eliminating the influence of previous surgical interventions. Additionally, those with severe chronic obstructive pulmonary disease (COPD) were excluded due to the potential influence on postprocedural recovery. Lastly, individuals with advanced chronic kidney disease, defined by an estimated glomerular filtration rate (eGFR) of <30 mL/min/1.73 m^2^, were excluded to reduce confounding variables related to renal dysfunction and its impact on cardiovascular outcomes.

### 2.3. Data Collection

Collected data included demographics, echocardiographic parameters (LVEF, TAPSE, PSAP), biochemical markers (hemoglobin, inflammatory markers, creatinine, ALAT, ASAT), systolic and diastolic blood pressure, and clinical outcomes.

Echocardiographic assessments were conducted utilizing the Philips iE33 ultrasound system (USA). Echocardiographic evaluations were performed using standardized protocols. Patients were positioned in a left lateral decubitus position to optimize acoustic window access. Standard 2D, M-mode, Doppler, and color flow imaging techniques were applied to assess TAPSE and PASP, respectively. Specifically, TAPSE was evaluated using M-mode echocardiography to quantify right ventricular systolic function, while PASP was determined through Doppler measurements, with accurate estimation of pulmonary hemodynamics. The TAPSE/PASP ratio was expressed in mm/mmHg. Patients were divided into two groups according to the median TAPSE/PASP ratio (cutoff = 0.32 mm/mmHg). Those with TAPSE/PASP ≤0.32 were classified as the low-ratio group, and those with TAPSE/PASP > 0.32 as the high-ratio group (Table 1).

The presence of atrial fibrillation (AF) was classified as paroxysmal, persistent, or permanent according to the ESC guidelines [27] and was recorded only if documented before the TAVR procedure (preprocedural AF). Primary outcome: new onset postprocedural AF (defined as any documented AF episode lasting ≥30 s). Hypertension was graded following the European Society of Hypertension classification (Grade 1: 140–159/90–99 mmHg; Grade 2: 160–179/100–109 mmHg; Grade 3: ≥180/≥110 mmHg) [28]. Secondary outcomes: all-cause in-hospital mortality, need for permanent pacemaker implantation, major vascular complications, acute kidney injury, and length of hospital stay. Complications were defined according to VARC-3 criteria [29].

### 2.4. Patient Grouping

Patients were categorized into two distinct groups based on their TAPSE/PASP ratio, allowing for a comparative analysis of right ventricular function and its impact on clinical outcomes. The division was based on the median TAPSE/PASP value, with one group consisting of patients with higher ratios, indicating a better RV function, and the other comprising those with lower ratios, suggestive of RV dysfunction (Table 1).

### 2.5. Statistical Analysis

Continuous variables were presented as mean ± standard deviation (SD) and compared using Student’s t-test or the Mann–Whitney U test. Categorical variables were expressed as frequencies and compared using the chi-square test or Fisher’s exact test. A *p*-value < 0.05 was considered statistically significant. Receiver-operating characteristic (ROC) curve analysis was performed to test the predictive value of TAPSE/PASP for postprocedural arrhythmia. AUC values were interpreted cautiously: 0.5–0.6 = poor, 0.6–0.7 = modest, 0.7–0.8 = acceptable, 0.8–0.9 = good, and >0.9 = excellent discrimination.

## 3. Results

### 3.1. Patient Demographics and Clinical Characteristics

A total of 100 patients were included in the study. The mean age was 77.8 years (95% CI: 76.65–78.97), with a standard deviation of 5.8373 years. The median hospital stay was 8.3 days (95% CI: 7.56–9.19), ranging from a minimum of 3 days to a maximum of 30 days. Demographic data analysis also indicated a balanced sex distribution (52% male, 48% female), suggesting an even representation of both genders.

The preexisting conditions showed that preprocedural arrhythmia was relatively low (6%), but postoperative arrhythmia showed a significant increase (38%), indicating that surgery may be a contributing factor. Previous stroke incidence was 12%, while new stroke occurrence was only 1%, suggesting a low rate of stroke events post-intervention. Coronary artery disease (26%) and carotid artery disease (12%) indicate a notable prevalence of vascular comorbidities in patients with severe AS. Diabetes mellitus was prevalent in 32% of individuals, a significant proportion with potential metabolic implications. (Table 2). Patients with lower TAPSE/PASP (≤0.32 mm/mmHg) had a higher NYHA class (III–IV in 71% vs. 45%, *p* = 0.013) and more severe hypertension (*p* = 0.021). PASP did not differ significantly between groups (47 ± 12 mmHg vs. 45 ± 11 mmHg, *p* = 0.47). TAPSE was markedly lower in the low-ratio group (15.8 ± 2.7 mm vs. 19.9 ± 2.9 mm, *p* < 0.001), confirming reduced RV systolic function as the main driver of group difference.

Medtronic valves were used in 74%, making them the predominant choice, while other valves accounted for 26%. All procedures were completed successfully with 100% device implantation. Self-expanding prostheses were used in 72%, and balloon-expandable in 28%. No annular rupture or coronary obstruction occurred; peri-procedural mortality was at 0%. Post-interventional complications were observed in 7% of cases, indicating a moderate postprocedural risk. Hematoma occurrence was 2%, indicating a relatively low postprocedural bleeding risk. New pacemakers were implanted in 9%, highlighting a need for postprocedural cardiac support.

Medication adjustments after TAVR were necessary in 18% of cases, reflecting the need for postprocedural pharmacological management, and refer to initiation, discontinuation, or dose change of beta-blockers, antiarrhythmics, or diuretics following TAVR. NYHA functional classification preprocedural analysis showed that most patients were stratified to Class II (66%), followed by Class III (29%), while severe symptoms (Class IV) were present in only 4%.

Arterial hypertension was highly prevalent, with Grade II being the most common (69%), followed by Grade III (26%) and Grade I (5%). Abdominal aortic aneurysms are present in 4%, indicating a lower but relevant risk group. A moderate prevalence of malignancies was reported (7%).

### 3.2. Subgroup Analysis

*Subgroup 1* (Lower TAPSE/PASP): exhibited a mean TAPSE/PASP ratio of 0.3607, with a 95% confidence interval (CI) ranging from 0.3322 to 0.3892. These patients had reduced right ventricular function and higher pulmonary pressure, indicative of a more compromised cardiovascular function (Table 1).

*Subgroup 2* (Higher TAPSE/PASP): patients in this category had a mean ratio of 0.5939, with a 95% CI between 0.5612 and 0.6266. The higher TAPSE/PASP ratio reflected a better right ventricular function and a more favorable pulmonary pressure profile, correlating with a higher degree of hemodynamic stability.

Subgroup 1 had a mean TAPSE/PASP ratio of 0.3607, while Subgroup 2 showed a higher mean ratio of 0.5939, with a difference between the two groups of 0.2332, with a statistically significant *p*-value of <0.0001, indicating a highly significant difference between the two groups. Patients in Subgroup 2 had better RV functions, as indicated by their higher TAPSE/PASP ratio, whereas patients in Subgroup 1 exhibited more impaired RV functions.

*General characteristics.* No statistically significant differences were observed in age, gender distribution, or hospital stay duration between the two groups (Table 3).

### 3.3. Pre- and Postoperative Atrial Fibrillation

**Figure 1:**
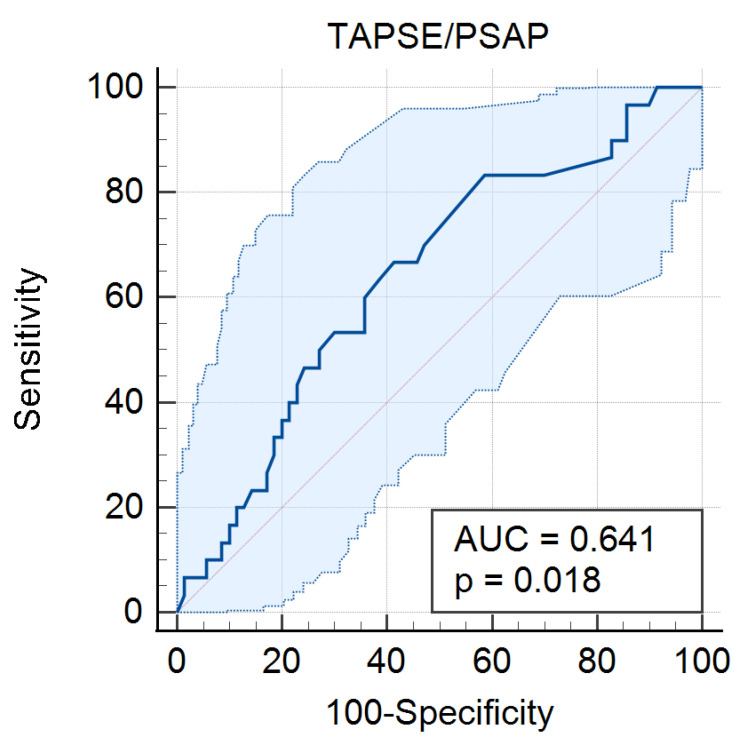
ROC curve showing predictive performance of TAPSE/PASP for post-procedural AF. The curve demonstrates moderate discriminatory ability (AUC = 0.641; 95% CI: 0.539 to 0.735, *p* = 0.018).

The patients in Subgroup 1 (lower TAPSE/PASP ratio) experienced a significantly higher incidence of postoperative atrial fibrillation (AF) compared to those in Subgroup 2 (higher TAPSE/PASP ratio) (48% vs. 28%, *p* = 0.044), suggesting an increased vulnerability to arrhythmic complications in patients with compromised right ventricular function and higher pulmonary pressures. In contrast, the incidence of preoperative AF did not differ significantly between subgroups (4% vs. 8%, *p* = 0.4021), with comparable baseline arrhythmia prevalence (Table 3). Receiver-operating-characteristic analysis showed that TAPSE/PASP modestly predicted post-TAVR AF (AUC = 0.634, 95% CI 0.526–0.743, *p* = 0.038). (Table 4, Figure 1).

The analysis of the echocardiographic findings before surgery revealed that patients in Subgroup 2 exhibited higher LVEF values (48.72% vs. 41.06%; *p* < 0.0001), indicating enhanced left ventricular function and potentially higher cardiac efficiency. LVEF improved modestly and similarly (41.1 ± 8.6% → 45.9 ± 7.5% vs. 49.5 ± 6.9% → 52.1 ± 6.3%, *p* interaction = 0.22). No significant difference in PASP was found post-procedure (46 ± 10 vs. 44 ± 9 mmHg, *p* = 0.36). Moreover, patients in Subgroup 2 exhibited higher TAPSE values (22.11 mm vs. 18.38 mm; *p* < 0.0001), indicating superior RV systolic function. On the other side, the pulmonary systolic arterial pressure (PASP) values were 50.08 mmHg in Subgroup 1 and 49.47 mmHg in Subgroup 2, with no statistically significant difference (*p* = 0.8446) between groups. This suggests that despite differences in RV function and contractility, pulmonary pressures remained comparable (Table 5).

In the evaluation of the comorbidities and complications, data analysis suggests that Grade III arterial hypertension was significantly more prevalent in Subgroup 2 (38%) compared to Subgroup 1 (14%), with a *p*-value of 0.0236, indicating statistical significance (Table 6). The occurrence of new strokes was documented at 2% in Subgroup 2, compared to 0% in Subgroup 1; however, the *p*-value of 0.3173 suggests that this difference does not reach statistical significance. The prevalence of coronary artery disease was recorded at 30% in Subgroup 1 and 22% in Subgroup 2, with a *p*-value of 0.3642, with no statistical significance.

In the analysis of comorbidities and complications, Grade III arterial hypertension was observed more frequently in Subgroup 2.

The comparative analysis between subgroups 1 and 2 revealed no significant differences for CAD prevalence and new stroke, but Grade 3 arterial hypertension was more prevalent in subgroup 2 (Table 6).

LVEF improved after surgery in both subgroups, but the differences were statistically significant only in the first subgroup (Table 7).

### 3.4. Biochemical Markers Before and After TAVR

Preprocedural measurements showed mean (alanine aminotransferase) ALAT levels of 28.24 U/L in Subgroup 1 and 25.16 U/L in Subgroup 2, with a mean difference of −3.08 U/L and a *p*-value of 0.2796, indicating no statistically significant difference between the groups (Table 8). Postprocedural measurements indicated ALAT levels of 24.80 U/L in Subgroup 1 and 30.98 U/L in Subgroup 2, with a difference of 6.18 U/L and a *p*-value of 0.4907, with no significant postprocedural variation (Table 8). No significant differences were also obtained for ASAT, diastolic blood pressure, creatinine, hemoglobin, leucocytes, neutrophils, and neutrophil-to-lymphocyte ratio (NLR).

The analysis of systolic blood pressure (BP) showed preprocedural mean systolic BP values of 126.04 mmHg in Subgroup 1 and 138.34 mmHg in Subgroup 2, with a mean difference of 12.30 mmHg and a *p*-value of 0.0027, indicating a statistically significant difference. Postprocedural measurements of systolic blood pressure indicated no statistically significant differences between Subgroup 1 and 2. However, the postprocedural values showed a non-significant change, suggesting comparable outcomes following intervention.

The hemoglobin levels remained consistent across both subgroups, with no statistically significant differences observed before or after the procedure. However, reductions in hemoglobin levels were observed post-TAVR in both groups, potentially due to procedural blood loss or hemodilution.

The analysis of lymphocyte levels showed preprocedural mean lymphocyte counts of 1.49 × 10^9^/L in Subgroup 1 and 1.57 × 10^9^/L in Subgroup 2, with a mean difference of 0.09 × 10^9^/L and a *p*-value of 0.3978, indicating no statistically significant difference. Postprocedural measurements recorded lymphocyte counts of 0.88 × 10^9^/L in Subgroup 1 and 1.11 × 10^9^/L in Subgroup 2, with a difference of 0.23 × 10^9^/L and a *p*-value of 0.0203, suggesting a statistically significant increase in Subgroup 2 post-procedure. These findings indicate that while preprocedural lymphocyte levels remained comparable between subgroups, a significant postprocedural increase was observed in Subgroup 2, possibly reflecting immune system activation in the context of the natural response to intervention. The significant drop in the lymphocyte counts postprocedural may suggest periprocedural stress responses.

## 4. Discussion

The present study evaluates the prognostic value of the TAPSE/PASP ratio in AS patients undergoing TAVR. The main finding was that a reduced TAPSE/PASP ratio (≤0.32 mm/mmHg) was associated with a higher incidence of postprocedural atrial fibrillation (AF), longer hospitalization, and poorer right-ventricular (RV) performance. Unlike previous analyses that considered TAPSE and PASP separately, this study confirms that their interaction provides additional clinical insight. In the context of AS, chronic LV outflow obstruction leads to pulmonary hypertension, elevated RV afterload, and ultimately RV dysfunction. Our findings are in line with earlier reports demonstrating that depressed RV–pulmonary coupling predicts poorer outcomes in both surgical and transcatheter valve populations. Several prior studies have examined the TAPSE/PASP ratio as a prognostic marker in valvular heart disease. In our cohort, the ratio’s AUC values around 0.63 are consistent with the modest predictive performance observed elsewhere and should therefore be interpreted as moderate discrimination rather than as a sensitive or specific test. Patients with lower TAPSE/PASP ratios exhibited significantly worse postoperative outcomes, including higher rates of AF recurrence, poorer RV function, and greater hemodynamic instability. These findings highlight the importance of preoperative RV function assessment in guiding clinical decisions and improving postoperative management [5,9,16]. Moreover, patients with impaired RV function often exhibit signs of right-sided heart failure, including peripheral edema and hepatic congestion [19]. These clinical signs, coupled with echocardiographic findings, require a multidisciplinary approach to optimize pre-TAVR hemodynamics through diuretic therapy, afterload reduction, and volume management.

Our study categorized patients into two subgroups. Subgroup 1 demonstrated weaker right ventricular function and increased pulmonary pressure, indicating a greater cardiovascular burden and potentially higher clinical risk, while Subgroup 2 exhibited better cardiac performance and more favorable pulmonary dynamics with greater hemodynamic stability and a less compromised cardiovascular state. The enhanced RV function observed in Subgroup 2 was a marker for a safer cardiac profile, correlating with improved clinical outcomes, better postoperative recovery, and lower cardiovascular risk. The inclusion of the patients into the two groups aimed to provide a better understanding of the clinical significance of TAPSE/PASP as an indicator of RV efficiency and heart-lung interaction and to assess its prognostic utility in periprocedural risk stratification in TAVR for AS. The higher TAPSE values reflect preserved RV systolic function, while the elevated LVEF indicates superior left ventricular performance, further reinforcing the significance of integrating TAPSE/PASP ratio assessment into preprocedural risk stratification protocols.

The analysis of patient characteristics revealed several critical observations regarding postprocedural arrhythmia and the preoperative incidence of diabetes, raising significant concerns, requiring longer monitoring and more complex management. Most patients fell within NYHA II–III, indicating moderate functional limitations that may impact overall quality of life and warrant appropriate clinical management. Hypertension, particularly Grade II, is highly prevalent. This higher prevalence suggests a potential improved RV adaptation to increased afterload in these patients, possibly indicating enhanced compensatory mechanisms that mitigate the impact of elevated arterial pressure. Diastolic blood pressure remained stable across both subgroups, with no statistically significant differences observed before or after the procedure. Baseline differences in NYHA class and hypertension severity may have influenced the observed associations. Although both groups were comparable for most clinical variables, these imbalances could partially explain outcome differences. Given the study’s retrospective design and limited sample size, multivariable adjustment was not feasible.

Encouragingly, the low incidence of new strokes and the relatively low complication rate suggest a favorable overall prognosis.

### 4.1. Postprocedural Arrhythmia Risk

A lower TAPSE/PASP ratio reflects impaired RV function and is linked to adverse clinical outcomes, including increased heart failure incidence, higher rates of postoperative arrhythmia due to increased electrical instability, and prolonged hospitalization [10]. This ratio serves as an independent prognostic parameter, whereas TAPSE or PASP alone do not provide significant prognostic value [20]. The TAPSE/PASP ratio is gaining recognition as a crucial prognostic marker for AF in TAVR patients. Current research has already demonstrated that a lower TAPSE/PASP ratio is independently associated with increased all-cause mortality, hospital readmissions, and a higher likelihood of requiring post-TAVR inotropic support [17]. Preprocedural identification of patients with a compromised TAPSE/PASP ratio can help stratify risk and guide periprocedural management strategies [18].

The analysis of preexisting conditions among the patients included in this study revealed a low incidence of preprocedural atrial fibrillation (AF) at 6%, while postprocedural arrhythmia increased significantly to 38%. ROC curve analysis revealed that PASP and TAPSE/PASP were predictors of postprocedural arrhythmias. This notable rise suggests that TAVR may be responsible for increased vulnerability to arrhythmic complications in patients with compromised right ventricular function and higher pulmonary pressures and that it may play a significant role in the development of arrhythmic events. However, the marked difference in postprocedural AF occurrence highlights the potential impact of RV dysfunction and pulmonary hemodynamics on periprocedural arrhythmic risk. These findings underscore the importance of preoperative risk stratification and the need for enhanced postprocedural monitoring and preventative strategies in patients with lower TAPSE/PASP ratios. Identifying patients at higher risk for AF could allow targeted interventions, such as rhythm control therapies, anticoagulation strategies, and optimized periprocedural management, to reduce complications and improve long-term outcomes following TAVR.

The significantly higher incidence of postprocedural atrial fibrillation (AF) in Subgroup 1 (48% vs. 28%, *p* = 0.0404) suggests that impaired right ventricular (RV) function, as indicated by lower TAPSE/PASP ratios, plays a key role in atrial electrical instability. This increased burden of AF can be attributed to multiple physiological mechanisms. Reduced RV contractility leads to elevated right atrial pressure and dilation, predisposing patients to AF through structural and functional remodeling of the atrial myocardium. Additionally, pulmonary hypertension, characterized by elevated PASP and poor RV adaptation, results in right-sided pressure overload, further exacerbating atrial remodeling and increasing the likelihood of AF recurrence. Neurohormonal activation also contributes to this heightened AF risk. Poor RV function stimulates the renin–angiotensin–aldosterone system (RAAS), which in turn promotes AF triggers and sustains arrhythmic substrates. The interplay between RAAS activation and oxidative stress plays a pivotal role in AF pathogenesis. Increased oxidative stress, mediated by enzymes such as NADPH oxidase, generates reactive oxygen species (ROS) that induce atrial oxidative damage, inflammation, and both electrical and structural remodeling, ultimately creating a substrate for AF development [21]. In conclusion, preprocedural identification of patients with lower TAPSE/PASP ratios could enable more tailored rhythm management strategies, more aggressive anticoagulation, early cardioversion, and closer postprocedural monitoring to lower the risk of sustained AF and associated complications. Understanding the relationship between RV function and AF incidence offers valuable insight into optimizing periprocedural care and improving patient outcomes [6,7].

### 4.2. Right Ventricular Function and Prognosis

TAPSE, a well-established marker of right ventricular (RV) systolic function, was found to be significantly lower in Subgroup 1 (18.38 mm vs. 22.11 mm, *p* < 0.0001), indicating preprocedural RV dysfunction. This impaired ventricular performance likely contributed to several adverse clinical outcomes. Patients with reduced TAPSE are at an increased risk of heart failure, as impaired RV contractility leads to inadequate cardiac output, resulting in venous congestion, peripheral edema, and prolonged hospital stays. Furthermore, poor RV adaptation delays recovery and rehabilitation, as persistent dyspnea and fatigue hinder functional improvement and extend the need for medical intervention. The prognostic significance of TAPSE is reinforced by evidence linking low TAPSE values to higher mortality rates in individuals undergoing cardiac procedures. This association underscores the critical role of RV function in determining postprocedural survival and long-term outcomes. Given these findings, preprocedural optimization of RV function is essential for high-risk patients. Although TAPSE showed the most significant difference between groups, PASP values were relatively similar. Nevertheless, the TAPSE/PASP ratio remains superior to TAPSE alone because it adjusts contractility for afterload, reflecting the efficiency of RV-PA coupling. Even in the setting of comparable PASP, differences in coupling ratio carry independent prognostic implications and have been validated as predictors of outcomes after TAVR. Targeted interventions such as diuretics, pulmonary vasodilators, and RV-supportive therapies may improve hemodynamic stability and enhance postprocedural recovery, ultimately contributing to better patient outcomes.

### 4.3. Hypertension as a Protective Factor?

A surprising result of this study is that hypertension can play the role of a possible protective factor. A higher prevalence of Grade 3 hypertension was observed in Subgroup 2 (38% vs. 14%, *p* = 0.0236), suggesting that chronic hypertension may play a role in facilitating better right ventricular (RV) adaptation to pressure overload. This finding implies that long-standing systemic hypertension could contribute to specific compensatory mechanisms that support RV function under stress. One possible explanation is compensatory myocardial hypertrophy, where prolonged exposure to elevated blood pressure triggers structural remodeling in the RV, enhancing contractility despite increased afterload. Additionally, preserved arterial compliance in hypertensive individuals might maintain adequate coronary perfusion, ensuring that the RV continues to function efficiently even in the presence of elevated pulmonary pressures. Although higher pulmonary pressures were initially interpreted as potentially protective, this association likely reflects preserved right ventricular systolic function. Elevated pulmonary pressures indicate that the right ventricle is still capable of generating adequate systolic pressure despite increased afterload, whereas in advanced RV dysfunction, pulmonary pressures decline due to reduced output and uncoupling. These observations underscore the complex relationship between hypertension and RV adaptation, highlighting the need for a personalized approach to hypertension management in AF patients undergoing TAVR. Striking the right balance between mitigating excessive afterload and preserving the potential benefits of improved RV adaptation will be crucial in optimizing patient outcomes.

### 4.4. Postprocedural Hemodynamic Changes

Postoperative declines in both systolic and diastolic blood pressures were observed in both groups, though the changes were more pronounced in Subgroup 1, suggesting that individuals with poorer right ventricular (RV) function were more vulnerable to hemodynamic instability following transcatheter aortic valve replacement (TAVR). Several physiological mechanisms may contribute to this heightened susceptibility. Additionally, fluid redistribution following valve replacement may disproportionately affect patients with pre-existing RV dysfunction, further exacerbating vascular load fluctuations. Moreover, persistently elevated PASP increases RV strain, limiting preload-reserve capacity and making effective cardiac adaptation more challenging. Given these risks, periprocedural hemodynamic support is essential for patients with lower TAPSE/PASP ratios. Optimized fluid management targeted inotropic therapy, and close hemodynamic monitoring may help mitigate post-TAVR instability, reducing complications and enhancing recovery. A tailored approach to managing RV dysfunction and pressure regulation could be pivotal in improving outcomes for this high-risk population.

### 4.5. Biochemical Markers and Systemic Stress

Postprocedural declines in hemoglobin and lymphocyte counts were observed in both groups, with more pronounced reductions in Subgroup 1, suggesting a heightened systemic inflammatory and hematologic response following transcatheter aortic valve replacement (TAVR). These changes reflect a complex interplay of periprocedural physiological stressors that may influence recovery and overall cardiovascular function. One contributing factor may be perioperative blood loss, where procedural bleeding and hemodilution lead to postprocedural anemia, potentially compromising oxygen delivery and prolonging recovery. Additionally, inflammatory stress plays a significant role, as elevated neutrophil counts and suppressed lymphocyte levels indicate systemic inflammatory activation, which may exacerbate RV dysfunction and delay postprocedural improvement.

The findings reveal that creatinine levels remained stable in both subgroups, with no statistically significant changes observed before or after the procedure.

Recognizing these effects underscores the importance of periprocedural optimization strategies aimed at mitigating these hematologic and inflammatory shifts. Iron supplementation, targeted anti-inflammatory therapies, and individualized postprocedural care may help enhance recovery and stability, particularly in patients with lower TAPSE/PASP ratios, who are more susceptible to hemodynamic instability.

In conclusion, this study highlights the TAPSE/PASP ratio as a crucial prognostic marker in patients with aortic stenosis undergoing transcatheter aortic valve replacement (TAVR). Several key findings reinforce its clinical significance: Patients with lower TAPSE/PASP ratios exhibited a markedly higher incidence of postprocedural AF, indicating increased susceptibility to arrhythmic complications. This underscores the importance of vigilant rhythm management and targeted interventions to mitigate postprocedural AF burden. Impaired RV function, reflected by lower TAPSE values, was associated with greater morbidity, prolonged hospitalization, and increased clinical vulnerability. These findings reinforce the need for preprocedural RV optimization to improve surgical outcomes. Interestingly, hypertension, particularly grade 3, emerged as a potential protective factor. Patients with higher TAPSE/PASP ratios and a history of long-standing hypertension demonstrated better postprocedural adaptation, suggesting that chronic hypertension may facilitate RV remodeling and pressure tolerance. Further research is warranted to explore this intriguing relationship. Additionally, hemodynamic challenges were significantly more pronounced in Subgroup 1, with notable postprocedural hypotension and instability. These findings highlight the necessity for tailored periprocedural support strategies, including optimized fluid management and targeted RV-supportive therapies to prevent postprocedural complications.

This study provides important contributions in the field of interventional cardiology and echocardiographic imaging and underscores the clinical importance of TAPSE/PASP assessment, advocating for its integration into preprocedural risk stratification and postprocedural management protocols to enhance patient outcomes. Incorporating TAPSE/PASP into the preprocedural assessment could improve patient risk stratification and guide individualized management. Patients with low ratios might benefit from closer hemodynamic monitoring, optimized diuretic therapy, and rhythm surveillance after TAVR. However, given the moderate discriminatory power, TAPSE/PASP should complement rather than replace existing risk models.

To optimize outcomes for AF patients undergoing TAVR, several targeted strategies should be implemented. Preprocedural risk stratification using TAPSE/PASP ratio assessment can help identify high-risk individuals, enabling tailored prehabilitation approaches that enhance RV function prior to surgery. Postprocedural monitoring should be prioritized for patients with lower TAPSE/PASP ratios, ensuring continuous rhythm surveillance and hemodynamic evaluation to mitigate arrhythmic and circulatory instability. Additionally, RV-directed therapies, including early administration of pulmonary vasodilators and optimized fluid management, may improve RV performance and support postprocedural recovery. Implementing these measures can lead to better surgical outcomes, reduced postprocedural complications, and enhanced long-term cardiovascular stability for AF patients undergoing TAVR. TAPSE/PASP has an important value where scores do not assess right ventricular function.

### 4.6. Future Directions

Further prospective studies are needed to explore the role of TAPSE/PASP-guided therapeutic interventions in AF patients undergoing TAVR, investigate the impact of pharmacologic RV support strategies on postoperative outcomes, and develop risk models incorporating TAPSE/PASP ratios to refine patient selection and procedural planning.

### 4.7. Study Limitations

While our study provides valuable insights into the relationship between the TAPSE/PASP ratio and outcomes in patients undergoing TAVR, several limitations should be considered. The study was conducted at a single institution, which may limit the generalizability of the findings to other populations with different demographic and clinical characteristics. The study’s retrospective design explains potential selection bias and missing data, which may affect the reliability of the findings. Prospective studies are needed to validate these results.

Echocardiographic Measurement Variability. TAPSE and PASP measurements are operator-dependent and subject to inter- and intra-observer variability, potentially impacting the accuracy and reproducibility of the results.

The absence of biomarkers such as NT-proBNP, cardiac troponin, and C-reactive protein restricts biochemical correlation; these markers should be included in future prospective analyses.

Lack of Long-Term Follow-Up. Our analysis focuses on short-term outcomes post-TAVR. However, the long-term prognostic value of the TAPSE/PASP ratio, particularly its impact on survival rates, functional improvement, and chronic cardiac performance, was not assessed in this study. Given its potential role in risk stratification and outcome prediction, future research will aim to explore these long-term implications, providing deeper insights into RV function dynamics, disease progression, and optimal management strategies for TAVR patients.

Further multicentric prospective studies are needed to further delineate the clinical utility of TAPSE/PASP-guided therapeutic interventions in AF patients undergoing TAVR, to incorporate the report into the preprocedural management algorithm. Future research should also focus on evaluating the effectiveness of pharmacologic RV support strategies in improving postprocedural outcomes for patients undergoing TAVR by assessing the role of inotropic agents, pulmonary vasodilators, and heart failure medications. Targeted RV therapy could also be a game-changer in improving hemodynamic stability, reducing arrhythmic complications, and shortening hospitalization duration in patients undergoing TAVR. Further integrating predictive risk models based on TAPSE/PASP ratios, clinicians may refine patient selection criteria and optimize procedural planning, ensuring that individuals with higher risk profiles receive tailored preprocedural and postprocedural management strategies. These advancements hold the potential to revolutionize personalized therapeutic approaches, enhance risk stratification protocols, and improve long-term prognoses for patients undergoing TAVR.

## 5. Conclusions

The TAPSE/PASP ratio represents a simple prognostic marker providing valuable insights into RV function and pulmonary pressure dynamics in patients with AS undergoing TAVR. Given its significance, preprocedural risk stratification becomes essential for identifying patients at higher risk of adverse outcomes and guiding personalized management strategies. In patients with severe AS undergoing TAVR, lower TAPSE/PASP ratios identify those at increased risk for postprocedural AF and longer hospitalization. Although predictive accuracy is modest, the index provides complementary information for periprocedural risk assessment. Future multicenter studies with larger cohorts and comprehensive biomarker evaluation are warranted to validate these findings and to refine threshold values for clinical use.

Patients with lower TAPSE/PASP ratios may exhibit compromised ventricular function and elevated pulmonary pressures, requiring enhanced preprocedural optimization, including hemodynamic monitoring, potential adjunctive therapies, and careful periprocedural planning. In contrast, those with higher ratios may demonstrate better right ventricular performance, possibly correlating with favorable postprocedural outcomes and a smoother recovery trajectory. Integrating TAPSE/PASP ratio assessment into routine preprocedural evaluation may enhance clinical decision-making, allowing for personalized interventions that optimize patient selection, procedural success, and long-term outcomes. Further multicentric studies are warranted to validate its prognostic value and refine risk-adjusted therapeutic strategies that improve overall survival and quality of life in AS patients undergoing TAVR. This study underscores the critical role of the TAPSE/PASP ratio as a prognostic marker in patients with aortic stenosis undergoing TAVR.

## Figures and Tables

**Table 1:** Patient Groups Categorized by TAPSE/PASP Ratio.

Parameter	Subgroup 1 (n = 50)	Subgroup 2 (n = 50)	Difference (95% CI)	*p*-Value
Mean TAPSE/PASP Ratio	0.3607 (95% CI: 0.3322–0.3892)	0.5939 (95% CI: 0.5612–0.6266)	0.2332 (95% CI: 0.1903–0.2760)	*p* < 0.0001
SD	0.103	0.118	–	–
Min–Max Values	0.0224–0.489	0.500–1.200	–	–

Abbreviations: TAPSE—Tricuspid Annular Plane Systolic Excursion; PASP—Pulmonary Artery Systolic Pressure; CI—Confidence Interval; *p*—*p*-value; SD—Standard Deviation.

**Table 2:** Patient demographics and clinical characteristics.

Characteristics	Value
Sex (Male/Female)	52%/48%
Preprocedural arrhythmia	6%
Postprocedural arrhythmia	38%
Previous stroke	12%
New stroke	1%
Coronary artery disease	26%
Carotid artery disease	12%
Complications	7%
Diabetes mellitus	32%
Hematoma	2%
New Pacemaker	9%
Medication adjustment	18%
NYHA I	1%
NYHA II	66%
NYHA III	29%
NYHA IV	4%
Medtronic valve	74%
Other valve types	26%
Hypertension Grade I	5%
Hypertension Grade II	69%
Hypertension Grade III	26%
Abdominal aortic aneurysm	4%
Neoplasms	7%

Abbreviations. NYHA: New York Heart Association Functional Classification; **%**: Percentage of total study population.

**Table 3:** General Characteristics of Subgroup 1 vs. Subgroup 2.

Characteristic	Subgroup 1	Subgroup 2	*p*-Value
Age	77.26 ± 5.1	78.36 ± 5.4	0.3487
Sex			0.4257
Male	24 (48%)	28 (56%)	
Female	26 (52%)	22 (44%)	
Preprocedural Atrial Fibrillation	2 (4%)	4 (8%)	0.4021
Postprocedural Atrial Fibrillation	24 (48%)	14 (28%)	0.0404
Previous Stroke	7 (14%)	5 (10%)	0.5403
New Stroke	0 (0%)	1 (2%)	0.3173
Coronary Artery Disease	15 (30%)	11 (22%)	0.3642
Carotid Disease	6 (12%)	6 (12%)	1.000
Complications	4 (8%)	3 (6%)	0.6966
Diabetes Mellitus	16 (32%)	16 (32%)	1.0000
Hematoma	1 (2%)	1 (2%)	1.0000
Pericardial effusion	5 (10%)	4 (8%)	0.7281
Medication Adjustment	10 (20%)	8 (16%)	0.6045
NYHA Class			0.0392
NYHA I	0 (0%)	1 (2%)	
NYHA II	27 (54%)	39 (78%)	
NYHA III	20 (40%)	9 (18%)	
NYHA IV	3 (6%)	1 (2%)	
Valve Type			0.1735
Medtronic Valve	34 (68%)	40 (80%)	
Other Valve Type	16 (32%)	10 (20%)	
Hypertension Grade			0.0236
Grade 1	3 (6%)	2 (4%)	
Grade 2	40 (80%)	29 (58%)	
Grade 3	7 (14%)	19 (38%)	
Abdominal Aortic Aneurysm	2 (4%)	2 (4%)	1.0000
Neoplasm	3 (6%)	4 (8%)	0.6966
**Mean Age (Years)**	77.26 (95% CI: 75.67–78.85)	78.36 (95% CI: 76.63–80.08)	0.3487
Mean Hospitalization Duration (Days)	8.70 (95% CI: 7.29–10.10)	8.06 (95% CI: 7.18–8.94)	0.4406

Abbreviations: NYHA: New York Heart Association Functional Classification; CI: Confidence Interval; *p*-value: Statistical significance value.

**Table 4:** Results of receiver-operating characteristic (ROC) curve analysis for TAPSE/PASP and postprocedural arrhythmias.

Classification Variable	State Variable	AUC (95% CI)	*p*
Postprocedural AF	TAPSE/PSAP	0.641 (0.539 to 0.735)	0.018
Postprocedural arrhythmias	TAPSE/PSAP	0.627 (0.524 to 0.721)	0.026
Postprocedural AF	PSAP	0.635 (0.533 to 0.729)	0.0256
Postprocedural arrhythmias	PSAP	0.636 (0.534 to 0.730)	0.0175

Abbreviations: CI: Confidence Interval; *p*-value: Statistical significance value, AUC: area under the curve.

**Table 5:** Echocardiographic Findings Before TAVR.

Parameter	Subgroup 1 (Lower TAPSE/PSAP)	Subgroup 2 (Higher TAPSE/PSAP)	*p*-Value
LVEF (%)	41.06	48.72	<0.0001
TAPSE (mm)	18.38	22.11	<0.0001
PASP (mmHg)	50.08	49.47	0.8446

**LVEF**: left ventricular ejection fraction; **TAPSE**: tricuspid annular plane systolic excursion; **PASP**: pulmonary systolic arterial pressure; **TAVR**: transcatheter aortic valve replacement; ***p*-value**: probability value indicating statistical significance (values < 0.05 are considered statistically significant).

**Table 6:** Comorbidities and Complications Associated with TAVR.

Condition	Subgroup 1 (%)	Subgroup 2 (%)	*p*-Value
Coronary Artery Disease	30%	22%	0.3642
New Stroke	0%	2%	0.3173
Hypertension (Grade 3)	14%	38%	0.0236

TAVR: Transcatheter Aortic Valve Replacement; *p*-value: Probability value indicating statistical significance (values < 0.05 are considered statistically significant).

**Table 7:** Left ventricular ejection fraction before and after surgery.

Subgroup	Variable	Before TAVR (95% CI)	After TAVR (95% CI)	*p*
Subgroup 1	LVEF	41.06 (38.073–44.047)	43.9 (41.172–46.627)	0.0009
Subgroup 2	LVEF	48.72 (47.144–50.295)	49.5 (48.24–50.759)	0.16

**Table 8:** Biological Comparisons of Subgroup 1 vs. Subgroup 2 Before and After TAVR Procedure.

Parameter	Preprocedural Subgroup 1 (95% CI)	Preprocedural Subgroup 2 (95% CI)	Difference (95% CI)	*p*-Value	Postprocedural Subgroup 1 (95% CI)	Postprocedural Subgroup 2 (95% CI)	Difference (95% CI)2	*p*-Value
ALAT	28.24 (23.40–33.08)	25.16 (22.18–28.14)	−3.08 (−8.71 to 2.55)	0.2796	24.80 (16.62–32.97)	30.98 (15.00–46.96)	6.18 (−11.61 to 23.98)	0.4907
ASAT	27.32 (23.37–31.27)	24.06 (20.74–27.38)	−3.26 (−8.36 to 1.84)	0.2077	42.73 (17.62–67.85)	31.54 (21.61–41.47)	−11.19 (−38.04 to 15.65)	0.4079
Systolic BP	126.04 (120.87–131.21)	138.34 (132.22–144.46)	12.30 (4.39 to 20.21)	0.0027	123.34 (118.42–128.26)	127.50 (123.09–131.91)	4.16 (−2.37 to 10.69)	0.2088
Diastolic BP	72.58 (69.43–75.73)	76.44 (72.58–80.30)	3.86 (−1.06 to 8.78)	0.1226	68.46 (65.00–71.92)	70.10 (66.98–73.22)	1.64 (−2.96 to 6.24)	0.4811
Creatinine	1.2524 (1.02–1.48)	1.0242 (0.94–1.11)	−0.2282 (−0.47 to 0.014)	0.0648	1.1922 (0.91–1.48)	1.0052 (0.90–1.11)	−0.1870 (−0.49 to 0.11)	0.2194
Hemoglobin	12.89 (12.43–13.36)	13.28 (12.83–13.73)	0.39 (−0.25 to 1.03)	0.2300	11.10 (10.60–11.59)	11.35 (10.90–11.81)	0.26 (−0.41 to 0.92)	0.4457
Leukocytes	7.08 (6.62–7.54)	7.00 (6.44–7.56)	−0.08 (−0.79 to 0.63)	0.8191	8.36 (7.76–8.97)	8.99 (8.16–9.82)	0.62 (−0.39 to 1.64)	0.2257
Lymphocytes	1.49 (1.36–1.62)	1.57 (1.42–1.73)	0.09 (−0.11 to 0.29)	0.3978	0.88 (0.77–0.98)	1.11 (0.94–1.27)	0.23 (−0.04 to 0.42)	0.0203
Neutrophils	4.99 (4.58–5.39)	4.75 (4.30–5.20)	−0.24 (−0.84 to 0.36)	0.4328	6.96 (6.34–7.58)	7.09 (6.26–7.92)	0.13 (−0.89 to 1.15)	0.7997
Neutrophil/Lymphocyte Ratio	3.78 (3.17–4.40)	3.35 (2.92–3.78)	−0.43 (−1.18 to 0.31)	0.2468	11.52 (8.13–14.90)	8.98 (5.92–12.05)	−2.54 (−7.05 to 1.97)	0.2672

ALAT: Alanine Aminotransferase (U/L); ASAT: Aspartate Aminotransferase (U/L); BP: Blood Pressure (mmHg); CI: Confidence Interval; TAVR: Transcatheter Aortic Valve Replacement; *p*-value: Probability value indicating statistical significance (values <0.05 are considered statistically significant).

## Data Availability

The data presented in this study are available on request from the corresponding authors.

## Abbreviations

The following abbreviations are used in this manuscript:

AF	Atrial Fibrillation
AS	Aortic Stenosis
CI	Confidence Interval
COPD	Chronic Obstructive Pulmonary Disease
eGFR	Estimated Glomerular Filtration Rate
LV	Left Ventricle
LVEF	Left Ventricular Ejection Fraction
NYHA	New York Heart Association (functional classification of heart failure)
PAH	Pulmonary Arterial Hypertension
*p*-value	Probability Value
PASP	Pulmonary Systolic Arterial Pressure
RAAS	Renin–Angiotensin–Aldosterone System
RV	Right Ventricle
SAVR	Surgical Aortic Valve Replacement
SD	Standard Deviation
TAPSE	Tricuspid Annular Plane Systolic Excursion
TAVR	Transcatheter Aortic Valve Replacement
